# Individualized muscle architecture and contractile properties of ankle plantarflexors and dorsiflexors in post-stroke individuals

**DOI:** 10.3389/fbioe.2024.1453604

**Published:** 2024-11-26

**Authors:** Ruoli Wang, Longbin Zhang, Hoor Jalo, Olga Tarassova, Gaia Valentina Pennati, Anton Arndt

**Affiliations:** ^1^ KTH MoveAbility Lab, Department of Engineering Mechanics, KTH Royal Institute of Technology, Stockholm, Sweden; ^2^ Biomechanics and Motor Control Laboratory, Swedish School of Sport and Health Sciences, Stockholm, Sweden; ^3^ Department of Clinical Sciences, Danderyd Hospital, Division of Rehabilitation Medicine, Karolinska Institutet, Stockholm, Sweden; ^4^ Department CLINTEC, Karolinska Institute, Stockholm, Sweden

**Keywords:** torque-angle relationship, torque-angular velocity relation, fascicle length, muscle thickness, pennation angle, ultrasound

## Abstract

**Objective:**

This study was to investigate alterations in contractile properties of the ankle plantar- and dorsiflexors in post-stroke individuals. The correlation between muscle architecture parameters and contractile properties was also evaluated.

**Methods:**

Eight post-stroke individuals and eight age-matched healthy subjects participated in the study. Participants were instructed to perform maximal isometric contraction (MVC) of ankle plantar- and dorsiflexors at four ankle angles, and isokinetic concentric contraction at two angular velocities. B-mode ultrasound images of gastrocnemius medialis (GM) and tibialis anterior (TA) were collected simultaneously during the MVC and isokinetic measurements. Individualized torque-angle and torque-angular velocity relations were established by fitting the experimental data using a second-order polynomial and a rectangular hyperbola function, respectively. Muscle structure parameters, such as fascicle length, muscle thickness and pennation angle of the GM and TA muscles were quantified.

**Results:**

Post-stroke subjects had significantly smaller ankle plantarflexor and dorsiflexor torques. The muscle structure parameters also showed a significant change in the stroke group, but no significant difference was observed in the TA muscle. A narrowed parabolic shape of the ankle PF torque-fiber length profile with a lower width span was also found in the stroke group.

**Conclusion:**

This study showed that the contractile properties and architecture of ankle muscles in post-stroke individuals undergo considerable changes that may directly contribute to muscle weakness, decreased range of motion, and impaired motion function in individuals after stroke.

## 1 Introduction

Skeletal muscles are considered as the biological force generator that is designed to provide the functional requirements for maintaining movement and posture (Dordević et al., 2014). In particular, ankle plantar- and dorsiflexor muscles controlling the ankle joints play an essential role in many daily activities (Hasson et al., 2011). The force developed by contracting muscle *in vivo* depends on the muscle length and velocity, with active and passive muscle forces contributing to total muscle force (Ward et al., 2009). Force-length and force-velocity relationships thus are commonly used to describe basic properties associated with force production capacity of a muscle. In practice, torque-angle (T−θ) and torque-angular velocity (T−ω) relationships were instead more often reported due to the advantages of readily direct measurement and no need of non-trivial assumptions of individual antagonistic muscle’s contribution. T−θ and T−ω relationship can provide a more comprehensive picture of various factors impacting muscle function at the joint level.

Stroke is one of the world’s leading causes of death and disability in adults (Aked et al., 2018; Shi et al., 2022). Post-stroke individuals often experience motor impairments dominantly on one side of the body, including muscle weakness, contracture, and spasticity (Hatem et al., 2016; Alamri et al., 2010). Ankle joint is one of the most affected lower-limb joints after stroke that about 60% of stroke individuals discharged from in-patient rehabilitation require an ankle orthosis and 34% of stroke individuals developing ankle contracture (Gao and Zhang, 2008; Chisholm and Perry, 2012; Hara et al., 2017). For many post-stroke individuals, the impaired dorsiflexors could lead to foot drop and increase the risk of stumbling and falling (Gorst et al., 2016). Moreover, weak and/or spastic plantarflexors may also reduce forward propulsion during gait (Vattanasilp et al., 2000; Zhang et al., 2013). Maximal isometric torque was often used in clinical settings as a marker of active muscle functional ability (Ramsay et al., 2014). However, peak torque often occurs at different joint angles for individuals due to variations in muscle-tendon properties, joint mechanics, and neuromuscular control (Kannus, 1991; Timmins et al., 2016). It is essential to take individual joint range of motion into consideration when evaluating muscle functionality in a non-intact group, i.e., post-stroke. It has been previously reported that age-related differences in contractile properties of both dorsi- and plantarflexors ( T−θ and T−ω relationships) were observed (Hasson et al., 2011). However, T−θ and T−ω relationships of ankle muscles have not been investigated in post-stroke individuals, which might yield new markers in joint functional ability.

Neurological impairment after stroke often leads to secondary skeletal muscle structural and functional alterations, which impact contractile properties of the muscle and force production capacity (Zhang et al., 2024). The macroscopic arrangement of muscle fibers is also highly influenced by different factors such as age, gender and diseases (Knarr et al., 2014; Körting et al., 2019). Various noninvasive techniques, including surface electromyography, near-infrared spectroscopy, and ultrasonography, are used to analyze and assess muscle parameters (Guo et al., 2022). Muscle structure parameters, such as fascicle length (FL), muscle thickness (MT) and pennation angle (PA) have been evaluated in post-stroke individuals *in vivo* using medical imaging techniques, e.g., ultrasonography (US). Schillebeeckx et al. (Schillebeeckx et al., 2021) conducted a systematic review and revealed significant architectural changes, including reduced muscle thickness and fascicle length in individuals after stroke. However, the relationship between these morphological changes and muscle weakness remains unclear and requires further investigation. Gao and Zhang (2008) reported shorter FL and smaller PA in gastrocnemius medialis (GM) at rest compared to a control group. Compared to the less-affected side, decreased FL was also observed in the GM and soleus on the paretic side, respectively (Zhao et al., 2015). For dorsiflexors, muscle volumes, muscle length, FL and PA were nevertheless found similar in tibialis anterior (TA) between paretic and non-paretic sides at neutral position (Ramsay et al., 2014). Although the authors stated that the muscular structure alteration was not the primary inhibitor of the dorsiflexor strength, it is unclear whether there are structural changes through joint range of motion in post-stroke individuals and how these changes are intertwined with force production capacity. Liu et al. (2014) reported that body weight support treadmill training can increase pennation angle and muscle thickness in the tibialis anterior and fascicle length in the medial gastrocnemius, suggesting that early rehabilitation can positively impact muscle structure and improve function in individuals after subacute stroke. To the best of our knowledge, only one study by Gao and Zhang (2008) has investigated the changes in contractile properties of the gastrocnemius muscle using an electrical stimulator. However, there is still a lack of knowledge on the contractile properties of paretic muscle in patients with neurological disorders, particularly under voluntary contraction conditions.

The purpose of the current study was therefore to quantitatively compare muscle architecture parameters, i.e., FL, PA and MT of GM and TA, and contractile properties ( T−θ and T−ω profiles) of ankle plantarflexors (PF) and dorsiflexors (DF) between post-stroke and non-disabled individuals. In addition, the relationship between the maximal isometric torque of the ankle and the fascicle length of GM and TA was also investigated, respectively.

## 2 Methods

### 2.1 Experimental setup

Eight hemiplegic post-stroke individuals (sex: 2F/6M; age: 53.5 ± 15.1 years; weight: 74.9 ± 13.5 kg; height: 174.5 ± 11.5 cm) were recruited from Department of Rehabilitation Medicine, Danderyd Hospital, Stockholm, Sweden. Inclusion criteria were: (1) stroke > 6 months prior to inclusion; (2) no anti-spastic treatment within 3 months; (3) absence of other lower limb injuries or disorders; (4) being able to walk with or without assistance. Eight non-disabled individuals (sex: 4F/4M; age: 53.1 ± 6.0 years; weight: 70.9 ± 13.3 kg; height: 175.5 ± 11.3 cm) without any history of neurological or musculoskeletal disorders were participated as a control group (HC). The study was approved by the Swedish Ethical Review Authority. All participants gave written informed consent according to the Declaration of Helsinki.

**FIGURE 1:**
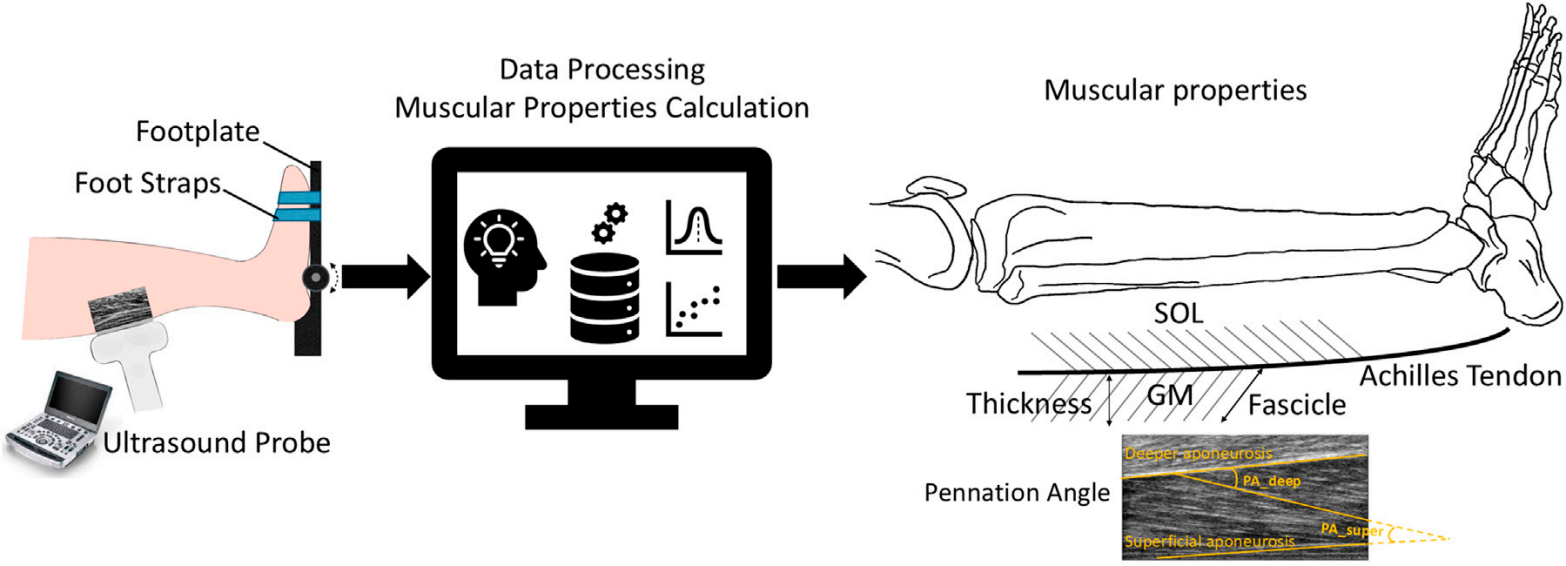
Overview of individualized muscle architecture and contractile properties estimation. Ultrasound images were recorded during maximal isometric and isokinetic concentric contractions. Muscle structure parameters such as fascicle length, muscle thickness, and pennation angle of the GM and TA muscles were quantified through data processing. $PA_{\mathrm{super}}$ and $PA_{\mathrm{deep}}$ refer to the angles formed between the fascicle and the superficial and deeper aponeuroses, respectively.

In order to measure T−θ and T−ω relationships of ankle PF and DF, each participant performed a series of isometric and isokinetic contraction on a dynamometer (IsoMed 2000, Hemau, Germany, Figure 1), while ankle torque and angle were recorded at 3 kHz. To quantify the muscle architecture parameters (FL, PA and MT), US images were recorded using an ultrasonography system (M9 Mindray, Shenzhen, China) with a 38 mm wide linear transducer (3.5–10 MHz). The torque and angle recordings were synchronized analogically and converted to digital data using Spike2 (Cambridge Electronic Design, United Kingdom). Raw data of the US were synchronized manually with other recordings using a trigger signal.

Participants performed the experiment in a seated semi-upright position with the knee flexed at 20° and the foot was fixated to a footplate of the dynamometer. Initially, the footplate was placed perpendicularly to the subject’s tibia, which was considered as the 0°ankle position, and the ankle joint was carefully aligned with the rotation axis of the dynamometer. Shoulders, hips, and legs were adequately strapped, and the tested foot was especially securely strapped to the footplate. The affected side was tested for the PS group and the right side was tested in the HC group, respectively. Prior to the testing, ankle range of motion (ROM) of each participant was determined and then passively rotated through the test ROM to familiarize the movement. A static gravitational compensation at the neutral position (ankle at 0°) was also applied following the user manual of the dynamometer. To determine the T−θ relation, each participant was instructed to perform maximal isometric contraction (MVC) of the ankle DF and PF at different ankle angles. For post-stroke individuals, testing angles were determined individually within their own ROM, typically between 5°dorsiflexion and 20°plantarflexion. All control participants performed MVC at 5°dorsiflexion, 0°neutral, 10°and 20°plantarflexion, which match the ROM of post-stroke participants. For each angle, two repeat isometric trials were performed with a duration of 5 s while having at least 30 s rest in between trials. The average of the two measurements was taken for further analysis. To determine T−ω relation, concentric isokinetic contractions were performed at two velocities: 60°/s and 90°/s with participants’ maximal efforts in five repetitions. Verbal encouragement from the investigator was provided throughout the measurement. During the MVC trials, US images were collected to quantify muscle structure parameters for GM and TA. The ultrasound transducer was optimally placed over the muscle belly in the sagittal plane, with sufficient transmission gel used for improved acoustic coupling (Balmaseda et al., 1986). The ultrasound probe was manually held by a trained operator, a licensed physician. The operator completed specialized sonography training at a dedicated ultrasonography clinic before the study. To ensure consistency, the same operator performed all the imaging for every participant.

### 2.2 Muscular properties calculation

T−θ : The averaged maximal isometric torque (TIM) measured at each ankle angle (θ) was used to construct the T−θ for each subject. The T−θ relationship was expressed as a second-order polynomial fit to the PF and DF TIM as a function of θ . The data fitting was performed using MATLAB’s Curve Fitting Toolbox. The maximum torque and angle of the polynomial curve were identified as the optimal isometric torque $T_{o}$ and optimal angle $\theta_{o}$ of each subject. For post-stroke individuals, $T_{o}$ and $\theta_{o}$ were identified within their own passive ROM.

T−ω profile: The concentric T−ω relationships were constructed from the peak isokinetic torque (TIV) and its corresponding angular velocity ω for each participant. The measured T−ω data was adjusted to account for the T−θ effects as suggested by previous studies (Caldwell et al., 1993; Hahn et al., 2014; Hasson et al., 2011; Lanza et al., 2003). The angle at which TIV occurred was identified, and the measured T−ω data point was scaled to the isometric torque produced at that angle. The scaled T−ω data was then fitted using a rectangular hyperbola Equation 1

$${\bar{T}}_{IV}=\frac{\left(1+A\right)B}{\omega +B}-A$$ (1)

where ${\bar{T}}_{IV}$ is the scaled isokinetic peak torque, and A and B are the fitting coefficients that describe the scaled T−ω relationship. ${\bar{T}}_{IV}$ at ω = 120°/s was extrapolated based on fitted rectangular hyperbola function.

Muscle architecture parameters: US images of GM and TA were analyzed using a previous custom-written MATLAB script (Körting et al., 2019). Firstly, the superficial and deep aponeuroses of each muscle were identified. Three visible muscle fascicles were then selected (the proximal, distal, and intermediate parts of the muscles). MT was calculated as the distance between the superficial and deep aponeuroses, and the average MT from these three regions (proximal, distal, and intermediate) was reported. The superficial and deep PAs were identified as the angles between the fascicle and the superficial aponeurosis and deeper aponeurosis, respectively. The average of the superficial and deep PAs was reported (Son et al., 2020; Körting et al., 2019). The fascicle length FL was calculated as the straight-line distance by dividing MT by the sine of the deep PA. Muscle architecture parameters were identified both at rest, and in MVC at the frame where maximal torque was observed at each angle.

$T_{\mathrm{norm}}-FL_{\mathrm{norm}}$ Profile (Normalized maximal isometric torque–Normalized fascicle length): The $FL_{\mathrm{norm}}$ is computed by normalizing the measured FL to the optimal fiber length, which is defined as the FL at the optimal angle. The measured FLs were first fitted with a first-order polynomial function; subsequently, the optimal FL was determined based on the fitted function. The width span was determined by calculating the range of $FL_{\mathrm{norm}}$ at 70% of maximal isometric torque.

### 2.3 Statistics

In order to take anthropometrical variance into consideration, TIM and TIV were normalized to the body weight, and FL and MT were normalized to the individual tibia length (the distance between the superior articular surface of the lateral condyle of the tibia to the tip of the medial malleolus). Spearman’s rank order correlation test was used to investigate the correlation between the optimal ankle torque and muscle structure parameters. Wilcoxon Mann-Whitney U test was used to investigate the group differences in normalized joint torque (TIM and TIV) and muscle parameters (FL and MT). Differences were considered statistically significant when p≤0.05 . All statistical analyses were performed using IBM SPSS Statistics (IBM Corp., Armonk, NY, United States). The fitness between the experimentally measured torque and curve-fitting function was computed using $R^{2}$ (Squared correlation coefficient). Due to the small dataset, we opted for a non-parametric statistical approach, reporting the median (min, max) for all values in the tables.

## 3 Results

### 3.1 Torque-angle measurement and T−θ profile

**FIGURE 2:**
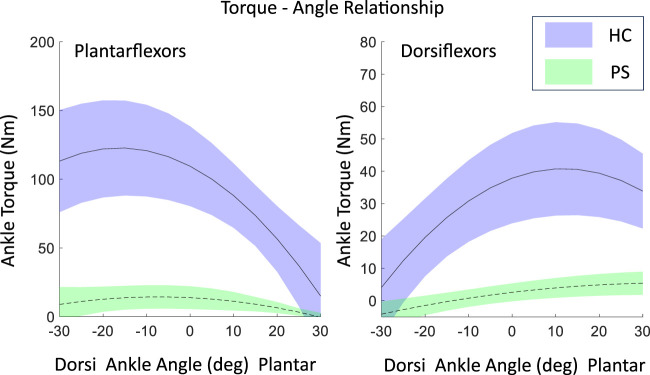
The maximal isometric contraction torque-angle relationship of ankle plantarflexors and dorsiflexion in both post-stroke individuals and healthy control groups, fitted using a second-order polynomial with experimental data, shown as mean ± standard deviation of all subjects.

Overall, post-stroke individuals showed a significant reduction in maximal isometric torque for both PF and DF across all tested ankle angles (Table 1), which also resulted in substantially smaller optimal isometric torque values for PF and DF. In the HC group, the optimal isometric torque values were 118.74 (75.70, 172.40) Nm for ankle PF and 32.87 (29.62, 67.94) Nm for ankle DF. The reduction in ankle torque in the PS group was substantial for both ankle PF [11.43 (2.39, 43.65) Nm], resulting in a reduction of 90.35%, and DF [6.23 (0.66, 32.77) Nm] with a reduction of 81.08%. In the HC group, both ankle DF and PF isometric T−θ showed a distinct parabolic shape. The optimal isometric torque was estimated at 11.86°(8.21°, 18.22°) plantarflexion for DF and at 20.23°(8.01°, 22.52°) dorsiflexion for PF. In contrast, in the PS group, the T−θ s for both DF and PF had a relatively flat shape. Notably, the optimal angle of ankle PF [14.38°(5.61°, 27.34°) dorsiflexion] was shifted from dorsiflexion to a slightly plantarflexed, while the optimal angle of ankle DF [28.02°(10.49°, 45.00°) plantarflexion] was shifted to a larger plantarflexed position (Figure 2). High correlations suggested a good curve fitting in the T−θ among all subjects (PS: PF $R^{2}$ = 0.97 ± 0.03, DF $R^{2}$ = 0.97 ± 0.04; HC: PF $R^{2}$ = 0.94 ± 0.05, DF $R^{2}$ = 0.96 ± 0.05).

**TABLE 1:** The measured isometric ankle torque (Nm) during isometric maximum voluntary Plantarflexion and Dorsiflexion contraction across subjects at four angle positions (5°dorsiflexion, 0°, 10°, and 20°plantarflexion) in both healthy control (HC) and post-stroke (PS) groups, presented as median (min, max). Values shown in bold indicate a statistically significant difference between the HC and PS groups based on Wilcoxon Mann-Whitney U tests.

Case	Plantarflexion		Dorsiflexion	
Position	HC	PS	HC	PS
5°dorsi	**117.4** **(60.1, 156.6)**	**7.7** **(2.0, 43.0)**	**28.2** **(22.8, 62.0)**	**3.0** **(0.2, 19.6)**
0°plantar	**110.0** **(66.0, 151.9)**	**9.8** **(1.9, 31.6)**	**30.4** **(13.9, 66.2)**	**1.9** **(0.2, 26.7)**
10°plantar	**77.8** **(48.8, 124.5)**	**7.8** **(0.3, 26.7)**	**32.2** **(9.4, 67.6)**	**3.5** **(0.6, 32.8)**
20°plantar	**64.6** **(38.4, 99.0)**	**2.3** **(0.04, 19.3)**	**31.7** **(28.5, 63.8)**	**6.0** **(0.6, 27.8)**

### 3.2 Torque-angular velocity measurement and T−ω profile

The scaled torque of the PF and DF showed no significant difference between groups at all tested velocities (Table 2, PF 60°/s: *p* = 0.46, 90°/s: *p* = 0.14; DF 60°/s: *p* = 0.52, 90°/s: *p* = 0.71). A significantly reduced torque in post-stroke individuals was found on the extrapolated data at 120°/s in DF (*p* = 0.02), but not in PF (*p* = 0.09).

**TABLE 2:** The scaled isokinetic peak ankle torque across subjects at three angular velocities (60°/s, 90°/s, and 120°/s (extrapolated)) in both healthy control (HC) and post-stroke (PS) groups, presented as median (min, max). Values shown in bold indicate a statistically significant difference between the HC and PS groups based on Wilcoxon Mann-Whitney U tests.

Case	Plantarflexion		Dorsiflexion	
Angular velocity (°/s)	HC	PS	HC	PS
60	0.64 (0.43, 0.78)	0.48 (0.25, 0.76)	0.59 (0.48, 0.73)	0.47 (0.11, 0.74)
90	0.53 (0.31, 0.70)	0.39 (0.14, 0.68)	0.48 (0.36, 0.63)	0.36 (0.04, 0.65)
120 (extrapolated)	0.45 (0.23, 0.63)	0.33 (0.07, 0.61)	**0.40** **(0.28, 0.56)**	**0.28** **(0.05, 0.58)**

**FIGURE 3:**
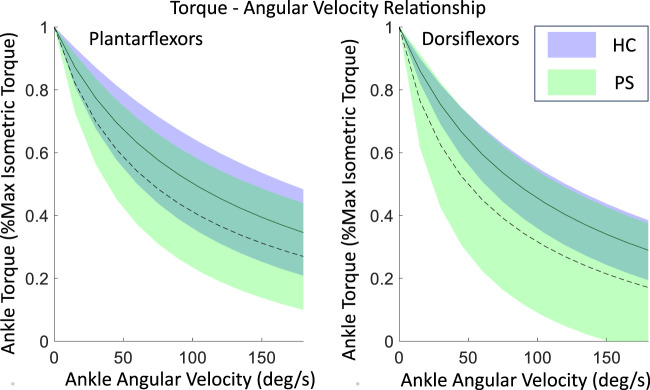
The maximal isometric torque-angular relationship of ankle plantarflexors and dorsiflexors in both post-stroke individuals and healthy control groups, fitted using a rectangular hyperbola function with experimental data. Shown as mean ± standard deviation of all subjects.

The shape coefficient A (Table 3), derived from the rectangular hyperbola function, was significantly different between the two groups in DF (*p* < 0.01) but not in PF (*p* = 0.20). The shape coefficient A resulted in a steeper decline at lower angular velocities in the PS group’s torque-angular velocity curve, compared to the HC group (Figure 3). No significant difference was found in the shape coefficient B in both PF (*p* = 0.23) and DF (*p* = 0.08).

**TABLE 3:** The shape coefficient value A and B, derived from fitting a rectangular hyperbola function to model ankle torque-angular relationships during isokinetic Plantarflexion and Dorsiflexion contractions across subjects in both the healthy control (HC) and post-stroke (PS) groups, presented as median (min, max). Values shown in bold indicate a statistically significant difference between the HC and PS groups based on Wilcoxon Mann-Whitney U tests.

Case	Plantarflexion		Dorsiflexion	
Variables	HC	PS	HC	PS
Shape Coefficient A	−0.40 (−0.89, 1.31)	−0.03 (−0.53, 0.28)	**−0.34 (-0.67, -0.02)**	**0.18 (-0.24, 0.74)**
Shape Coefficient B	0.51 (0.18, 4.38)	1.70 (0.12, 2.06)	**0.56 (0.03, 1.94)**	**1.60 (0.33, 2.06)**

### 3.3 Muscular architecture properties

**FIGURE 4:**
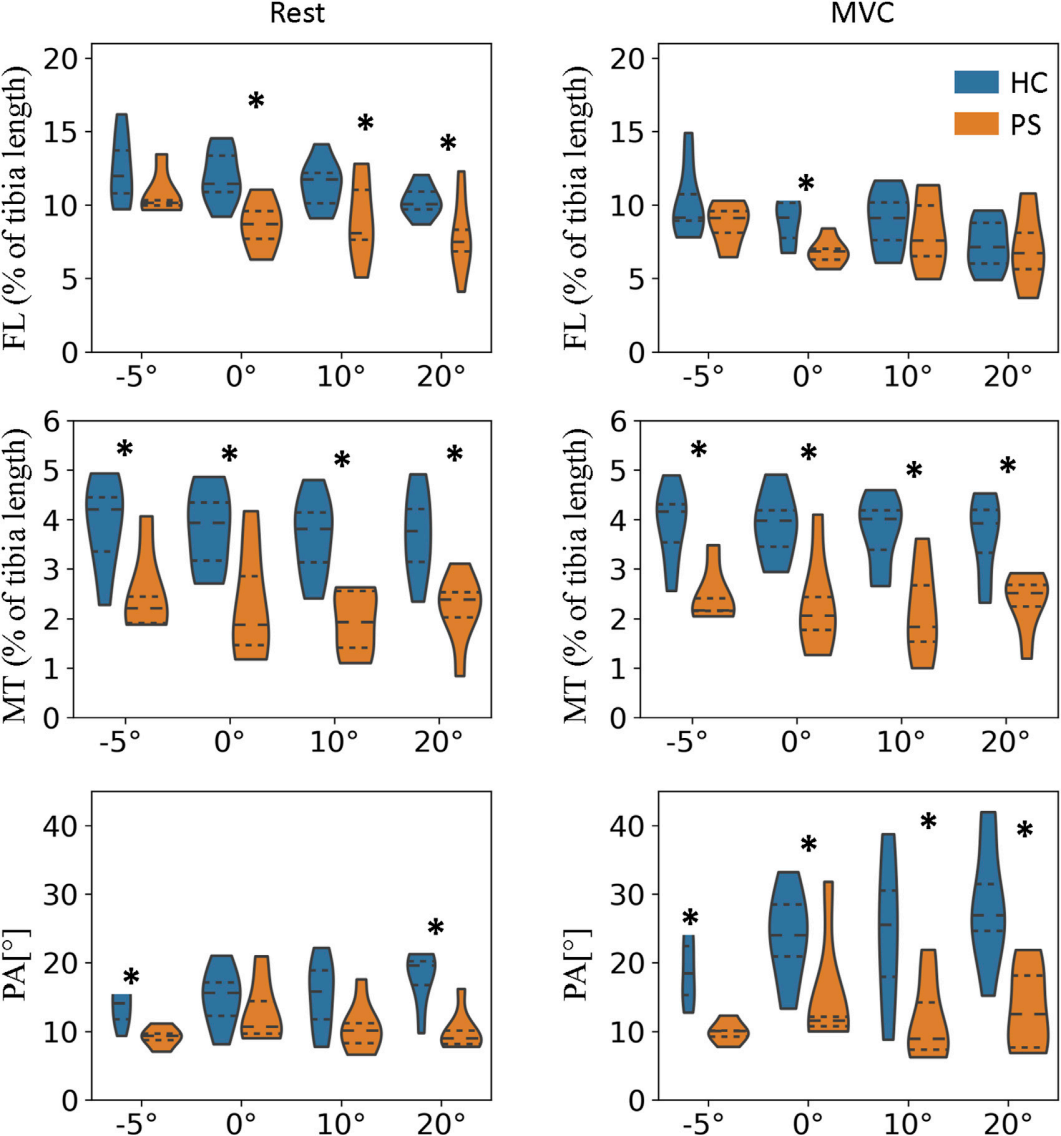
Fascicle length, muscle thickness, and pennation angle of gastrocnemius medialis (GM) in both post-stroke individuals and healthy control groups. ∗ indicate a statistically significant difference between the HC and PS groups based on Wilcoxon Mann-Whitney U tests.

At rest, the normalized FL of GM in the PS group is significantly shorter than in the HC at neutral position and 10°, and 20° of plantarflexion (Figure 4; 0°: p=0.01 ; 10°: p=0.02 , 20°: p=0.04 ); and somewhat, though not significantly, also shorter at 5°dorsiflexion (p=0.18) . Similar FL was observed in both groups at MVC, with only an exception of significantly shorter FL at the neutral position (p=0.03) .

The MT of GM was found significantly smaller in the PS group at all tested angles in both rest and MVC (Figure 4; Rest −5°: p=0.02 , 0°: p=0.03 , 10°: p<0.01 , 20°: p<0.01 ; MVC -5°: p<0.01 , 0°: p=0.02 , 10°: p<0.01 , 20°: p=0.02 ).

The PA of GM was found significantly smaller in the PS group at all tested angles in MVC (Figure 4; −5°: p<0.01 , 0°: p=0.03 , 10°: p=0.01 , 20°: p<0.01 ); At rest, significantly smaller PA was found at 5°dorsiflexion (p<0.01) and 20°plantarflexion (p<0.01) ; and somewhat, though not significantly, lower at 0° (p=0.28) and 10° (p=0.07) plantarflexion.

**FIGURE 5:**
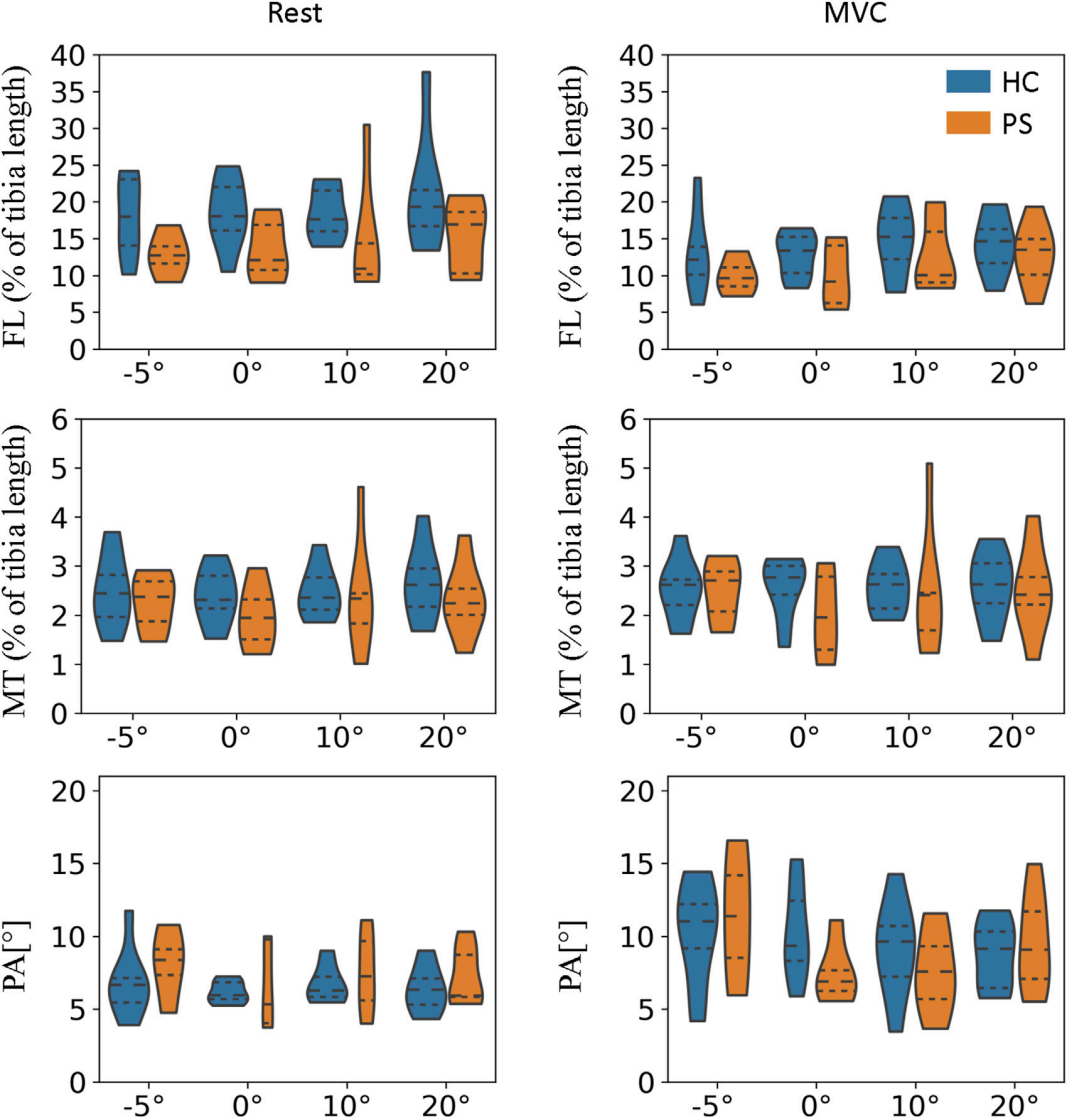
Fascicle length, muscle thickness, and pennation angle of tibialis anterior (TA) in both post-stroke individuals and healthy control groups.

For TA, the FL at rest was somewhat shorter in the PS group than in the HC group; however, the differences were not significant. The differences in FL at MVC were almost non-evidently. In addition, no significant differences were found in the MT and PA in any of the tested ankle angles (Figure 5).

### 3.4 Normalized isometric torque–normalized FL profile

**FIGURE 6:**
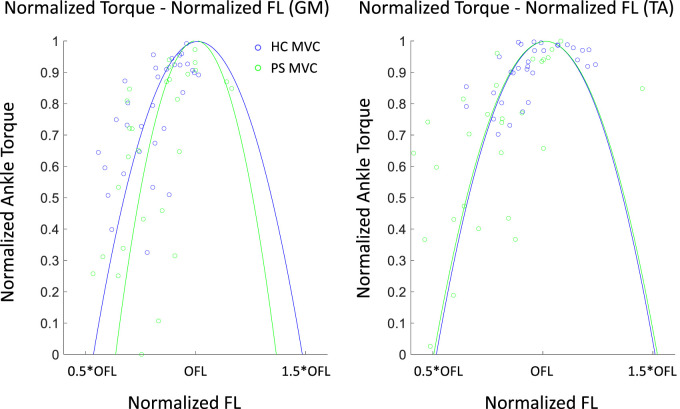
Normalized Ankle plantarflexor (GM) and dorsiflexor (TA) torque–normalized fascicle length profile in both post-stroke individuals and healthy control groups. An individual torque - length curve was fitted for each subject, and the mean of all fitted curves is shown here. The dots illustrated the measured values. OFL: optimal fiber length.

In the PS group, the ankle PF $T_{\mathrm{norm}}-FL_{\mathrm{norm}}$ profile had a narrower parabolic shape and a smaller width span compared to the HC group (Figure 6; Table 4). In both groups, the measured torques fell mostly in the ascending arm of the profile. Compared to controls, however, the normalized torque was more sparsely distributed, spanning from 0% to the maximal ankle torque. The shape of ankle DF $T_{\mathrm{norm}}-FL_{\mathrm{norm}}$ profile is comparable between groups (Figure 6) with a similar width. However, in the HC, the measured torque is mostly centered around the optimal fiber length, whereas in the PS group, it is scattered across the entire ascending arm of the profile. Compared to the HC group, the optimal fascicle length was found significantly shorter in the GM in the PS group (Table 4; p=0.03 ), but was similar in the TA (p=0.96) .

**TABLE 4:** The optimal fascicle length (OFL) and width span (%OFL) across subjects in both healthy control (HC) and post-stroke (PS) groups, presented as median (min, max), derived from ankle maximal Isometric Torque–Fascicle Length Profile. Values shown in bold indicate a statistically significant difference between the HC and PS groups based on Wilcoxon Mann-Whitney U tests.

Case	Gastrocnemius medialis		Tibialis anterior	
	HC	PS	HC	PS
Optimal Fascicle Length (mm)	**45.47** **(36.02, 56.82)**	**38.96** **(32.48, 46.27)**	53.26 (44.95, 93.08)	67.68 (33.60, 82.63)
Width Span (%OFL)	51 (33, 85)	46 (18, 64)	51 (33, 115)	48 (28, 99)

## 4 Discussion

In this work, we investigated contractile properties ( T−θ and T−ω ) and muscle architecture parameters of the major ankle dorsi/plantarflexors in post-stroke individuals and healthy controls during maximal isometric contraction. Compared with healthy controls, post-stroke subjects had significantly weaker ankle muscles in terms of decreased optimal torque in both PF and DF. Compared to controls, a significantly different T−ω shape can lead to a diminished torque production capacity at a higher contraction velocity in DF. Significant muscle architecture adaptations, including shorter FL, reduced MT, and smaller PA, were observed in GM. Additionally, a narrowed parabolic shape of the ankle PF $T_{\mathrm{norm}}-FL_{\mathrm{norm}}$ profile with a lower width span was also noted. Our findings indicated that both impaired muscle force generation capacity and muscle structural changes contributed to the functional limitation at the ankle joint level after stroke.

Muscle weakness, defined as the inability to generate normal levels of torque during an MVC is a dominant deficit after hemispheric stroke. The severity of the muscle weakness has been approved to be one of the primary factors associated with physical limitation and may account for 66%–72% of the variation in the temporo-spatial parameters of gait after stroke (Kim and Eng, 2003). Ankle PF were the major contributor to forward progression (Neptune et al., 2001) and the strength of DF was reported to have the strongest association with gait velocity in the paretic side following stroke (Neptune et al., 2001). However, muscle strength of these two muscle groups was less commonly reported in post-stroke group, especially in DF. Compared to controls, significantly reduced maximal dorsiflexion and plantarflexion torque were observed in all tested angles in the current study. Similar observations were reported by Dias et al. (2017) and Kowal et al. (2020) while the ankle joint was only assessed in the neutral position. Son and Rymer (2021) investigated the maximal isometric plantarflexor torque at several joint angles on both sides of post-stroke subjects and also found significantly smaller torque on the paretic side. No previous study has reported maximal isometric dorsiflexion torque across joint angles as in the current study.

Evaluating the MVC T−θ relationship provides an in-depth understanding of muscle weakness after stroke, such as whether it manifests at specific joint angles and identifying the optimal movement range. This information can be valuable for designing effective strength training interventions. Compared with the control group, the reduction of torque on the paretic side was larger at joint angles where muscle length is shortened, i.e., when the ankle was in a dorsiflexion position for PF, which was previously described as a selective muscle weakness after stroke (Ada et al., 2003). Similar observations were reported in elbow (Ada et al., 2003) and knee muscles (Horstman et al., 2009). In this study, we observed that due to the generally flat shape of the T – θ curve, we were unable to determine the optimal dorsiflexion angles within the tested ROM in the post-stroke group. Additionally, the optimal angle for PF in the control group fell outside the measured range. After interpolation, the optimal angle of PF was estimated at a less dorsiflexion position (14.38°) and the optimal angle of the DF also shifted to a more plantarflexion angle (28.02°). This shift may be attributed to the limited dorsiflexion ROM after stroke, which could place the ankle muscles in a more plantarflexed position, offering an advantage for force production. In the control group, aligned with the literature, the operating range of PF is located mostly at the descending limb of the curve, while DF located mostly at the ascending limb (Figure 2). The estimated optimal angle for PF was 20.23°dorsiflexion and 11.86°plantarflexion for DF, which were also in agreement with the literature (Paterson et al., 2007). However, it should be noted that these values are extrapolated rather than directly measured and should, therefore, be interpreted with caution. Despite this, the alignment of trends across groups with the literature (Paterson et al., 2007) supports the plausibility of these estimates. These findings may provide important guidance for rehabilitation interventions, which have been shown to improve muscle structure and function after stroke. Identifying the selective weakness at specific joint angles, particularly in the muscle’s shortened range (Ada et al., 2003), allows for more targeted strength training interventions by clinicians. These interventions can enhance force production in areas where muscle weakness is most pronounced, thereby improving rehabilitation outcomes (Liu et al., 2014; Ada et al., 2003; Sharp and Brouwer, 1997).

Compared to eccentric contraction, concentric torque production was reported more significantly impaired after stroke. It was shown that PF of the paretic side had a greater reduction in concentric torque than DF at an angular velocity of 30°/s, while eccentric torque had better preservation in both muscle groups (Eng et al., 2009). Kowal et al. (2020) also reported that compared to controls, significantly decreased plantarflexion torque at even higher concentric angular velocities, but no significant differences were found in DF. However, most studies only evaluated at one angular velocity and the overall T−ω relationship has not been previously presented in subjects after stroke. It was worth mentioning that we adopted a scaling approach (Lanza et al., 2003) to account for the shape of the underlying T−θ relationship in the determination of the T−ω profile. In other words, the scaled torque was determined by scaling the peak torque generated at one angular velocity to the maximal isometric torque at the same angle producing that peak torque. Therefore, the T−ω relationship is independent of the optimal isometric torque $T_{o}$ and can be characterized by two shape parameters A and B, which correspond to the dynamic constants of the force-velocity equation of the Hill-type muscle model (Alcazar et al., 2019). As suggested by Hasson et al. (2011), the independently increasing A led to a lower torque at any given concentric velocity, while increasing B can lead to a higher concentric but lower eccentric torque. The shape of the T−ω profile was found similar for PF in two groups (Figure 3) but significantly differed in DF with a weaker/slower concentric force (smaller B) in the post-stroke group. The observed differences in the T−ω profile imply the changes in the intrinsic force-velocity properties of the DF following stroke, which could be related to secondary changes in the size and/or number of the fast-twitch muscle fibers in the TA. After a stroke, the number of Type II fibers was found to progressively decrease, which contributed to the diminished force production and contraction speed, consequently impacting negatively daily mobility (Tieland et al., 2018).

Muscle structure adaptation is another factor impacting on the contractile properties of muscle post-stroke. In the rest, significantly shorter fascicle length was observed in the GM in all tested angles except 5 °dorsiflexion, which was aligned with previous findings (Dias et al., 2017). However, Gao and Zhang (2008) also reported the differences were more pronounced in dorsiflexion positions, contrary to our observation. The discrepancy was most probably due to missing data in the post-stroke group that three participants were not able to reach any dorsiflexion position. Compared to controls, no difference in fascicle length was found in TA across all joint angles. Under maximal contraction, muscle fascicle length of both GM and TA did not differ between the paretic side and healthy participants. It indicated that larger fascicle excursion of GM (length differences under the rest and MVC) occurred in healthy muscle and therefore led to a similar length as paretic muscle under the maximal contraction. Similar findings were also reported by Dias et al. (2017). This observation highlighted the importance of restoring fascicle length and range of motion during rehabilitation intervention because the fiber excursion is generally proportional to fiber length (Dias et al., 2017).

The joint torque and fascicle length relationship depicted the association of muscle function at the joint level and muscle’s intrinsic structural property. The ankle range of motion in able-bodied subjects was generally accepted as 50°plantarflexion to 20°dorsiflexion. This range corresponds to the whole ascending limb and a very small portion of the descending limb of the plantarflexion torque and fascicle length relationship in GM. It benefits biomechanically with a rather low passive force and potentially prevents injury from overstretching. This phenomenon may be explained by the fact that muscle passive force is relatively low within this range. Previous studies have evaluated the contractile properties of GM by applying electrical stimulation (Gao and Zhang, 2008). Similar active force-fascicle length and torque-angle behavior were reported. In the present study, the actual measured ROM covered approximately from 30% to the maximal isometric torque on the ascending limb of the torque–fascicle length profile. A similar distribution can be found in the post-stroke group. However, the width span of the torque-fascicle length relationship of GM in the post-stroke group was somewhat narrower and the slopes of the ascending and descending limbs were also steeper. The narrowed width span suggests that post-stroke participants may have a somewhat restricted range in fascicle length excursion during contraction, potentially leading to a limited range of motion at the joint level during daily activities. Further investigation on muscle sarcomere level may provide a further in-depth understanding of fascicle excursion restriction in the post-stroke muscles (Adkins et al., 2022).

The clinical presentation of post-stroke patients is usually a combination of weakness and other symptoms such as spasticity and contractures that are impacted by the muscle architectures and mechanical properties; therefore, comprehensive treatment requires strategies taking multiple factors into consideration as highlighted in the current study. Systematic reviews and studies showed that strength can be improved by progressive resistance training in persons after a stroke (Ouellette et al., 2004; Ada et al., 2006; Flansbjer et al., 2012). These strength improvements are also correlated with increasing muscle function and reducing limb disability. In addition, the effect of a stretching program on spastic PS subjects was investigated by other studies (Saunders et al., 2020). It was found that the reduction of spasticity and muscle stiffness are associated with the increased FL. Therefore, the integration of our research findings into post-stroke rehabilitation interventions holds significant promise for enhancing outcomes related to spasticity and mitigating secondary complications such as weakness and changes in muscle architecture.

We acknowledge that a 38 mm wide transducer might not be able to fully visualize the fascicles in the posterior and distal part of the muscle, therefore linear extrapolation was performed. The fascicles in the middle part of the muscle are commonly intact within the field of view. However, we did not expect a large error due to the extrapolation because gastrocnemius medialis and tibialis anterior muscles exhibit less curvature compared to the biceps femoris (Franchi et al., 2020). We investigated muscle architecture and contractile properties in post-stroke individuals and we acknowledge that factors beyond muscle morphology also contribute to strength loss in the post-stroke. Impaired motor unit recruitment, reduced firing rates, and changes in motor control strategies are known neural factors that significantly affect force generation, independent of muscle size or architecture (Hu et al., 2016).

Due to time constraints and participant safety considerations, concentric contractions were only performed at 60°/s and 90°/s. This restriction reduced our ability to interpret the inter-group difference at a higher extrapolated angular velocity, i.e., 120°/s. However, although it was not directly measured, our observation aligns with findings from Clark et al. (2006), who observed a pronounced velocity-dependent impairment in concentric knee torque production at higher velocities. This trend suggests a consistent response in torque-velocity relationships across studies, likely due to impaired agonist activation and reduced neural drive during faster movements. Authors also acknowledge that future studies including multiple additional high-velocity measures if feasible would provide a complete picture of the velocity-dependent impairments in individuals.

It’s also important to note that we only investigated the muscle architecture of TA from ankle dorsiflexors and GM from ankle plantarflexors. This restriction stems from the limitation that ultrasound can only accurately assess superficial muscles. We analyzed an overall torque/force-length profile for all plantarflexors and dorsiflexors, without distinguishing individual muscles. Future research employing techniques such as simulation could enable the decomposition of torque to individual muscle force, allowing for a direct examination of force/length characteristics in specific muscles. In addition, we only tested on the affected leg and two concentric contraction velocities in the post-stroke individuals to avoid fatigue due to already extensive data acquisition protocol. Future research, with a larger cohort exploring muscle architecture and contractile properties on both paretic and non-paretic sides, can provide a comprehensive understanding of muscle function and mobility after stroke, enhancing the generalizability of findings.

## 5 Conclusion

Stroke is a neurological disorder that can lead to muscle weakness and other secondary muscle morphological changes affecting joint function and overall mobility. We investigated the effects of stroke on major ankle joint muscles by examining their active contractile properties (specifically, the T−θ and T−ω relations) and architecture parameters during maximal isometric and isokinetic contractions. Both ankle plantarflexors and dorsiflexors were found significantly weaker in individuals after stroke. The muscle architecture parameters were significantly affected in GM, though not in TA. Additionally, by depicting the normalized torque-fascicle length profile, the effective FL operating range and torque generation capacity of each individual can be further illustrated. In conclusion, this study presented a quantitative evaluation of contractile properties of ankle muscles with a multi-level factors approach. Our findings yield important insights into the mechanisms of muscle weakness and motor impairments after stroke, contributing to the development of more effective rehabilitation interventions.

## Data Availability

The original contributions presented in the study are included in the article/supplementary material, further inquiries can be directed to the corresponding author.
